# Microscopic Inspection of the Adhesive Interface of Composite Onlays after Cementation on Low Loading: An In Vitro Study

**DOI:** 10.3390/jfb14030148

**Published:** 2023-03-07

**Authors:** Tiago Magalhães, Rita Fidalgo-Pereira, Orlanda Torres, Óscar Carvalho, Filipe S. Silva, Bruno Henriques, Mutlu Özcan, Júlio C. M. Souza

**Affiliations:** 1 University Institute of Health Sciences (IUCS), CESPU, 4585-116 Gandra PRD, Portugal; 2 Center for Interdisciplinary Research in Health (CIIS), Faculty of Dental Medicine (FMD), Universidade Católica Portuguesa (UCP), 3504-505 Viseu, Portugal; 3 Oral Pathology and Rehabilitation Research Unit (UNIPRO), University Institute of Health Sciences (IUCS), CESPU, 4585-116 Gandra PRD, Portugal; 4 Centre for MicroElectromechanical Systems (CMEMS-UMINHO), Campus Azurém, University of Minho, 4800-058 Guimarães, Portugal; 5 LABBELS Associate Laboratory, University of Minho, 4710-057 Braga, Portugal; 6 Ceramic and Composite Materials Research Group (CERMAT), Department of Mechanical Engineering (EMC), Federal University of Santa Catarina (UFSC), Florianopolis 88040-900, Brazil; 7 Division of Dental Biomaterials, Center of Dental Medicine, Clinic of Reconstructive Dentistry, University of Zurich, 8032 Zurich, Switzerland

**Keywords:** onlay, resin composite, cementation, resin cement, dentin

## Abstract

Purpose: This study aimed to assess the layer thickness and microstructure of traditional resin-matrix cements and flowable resin-matrix composites at dentin and enamel to composite onlay interfaces after cementation on low loading magnitude. Materials and Methods: Twenty teeth were prepared and conditioned with an adhesive system for restoration with resin-matrix composite onlays manufactured by CAD-CAM. On cementation, tooth-to-onlay assemblies were distributed into four groups, including two traditional resin-matrix cements (groups M and B), one flowable resin-matrix composite (group G), and one thermally induced flowable composite (group V). After the cementation procedure, assemblies were cross-sectioned for inspection by optical microscopy at different magnification up to ×1000. Results: The layer thickness of resin-matrix cementation showed the highest mean values at around 405 µm for a traditional resin-matrix cement (group B). The thermally induced flowable resin-matrix composites showed the lowest layer thickness values. The resin-matrix layer thickness revealed statistical differences between traditional resin cement (groups M and B) and flowable resin-matrix composites (groups V and G) (*p* < 0.05). However, the groups of flowable resin-matrix composites did not reveal statistical differences (*p* < 0.05). The thickness of the adhesive system layer at around 7 µm and 12 µm was lower at the interfaces with flowable resin-matrix composites when compared to the adhesive layer at resin-matrix cements, which ranged from 12 µm up to 40 µm. Conclusions: The flowable resin-matrix composites showed adequate flowing even though the loading on cementation was performed at low magnitude. Nevertheless, significant variation in thickness of the cementation layer was noticed for flowable resin-matrix composites and traditional resin-matrix cements that can occur in chair-side procedures due to the clinical sensitivity and differences in rheological properties of the materials.

## 1. Introduction

Dental restorations, such as crowns or onlays, can be performed by using chair-side clinical techniques or laboratorial procedures [1,2,3,4]. The type of dental restoration is dependent on several factors related to the tooth damage, remanent tooth tissues, aesthetic outcomes, and patient-related conditions [2,5,6]. For instance, the damage of teeth involving cusps determines the indication for onlay restorations concerning the degree of loss of tooth structures [6,7,8,9]. Prosthetic structures, such as onlays, are cemented with resin-matrix cements, which must be polymerized to guarantee the retention of the restoration. However, a progressive degradation of the restorative interfaces and a propagation of cracks can take place due to the negligence on the polymerization of the luting materials. The presence of cracks leads to the catastrophic fracture at the interface and detachment of the restorative materials.

The designing and the manufacturing of dental restorations has been increasingly carried out by computer-assisted design/computer assisted manufacturing (CAD-CAM) [10,11,12,13,14]. First, the digital scanning is performed, then the CAD system allows to designing the maxillofacial relationship, occlusal plane orientation, tooth mold, fitting, and the selection of shade and colors [10,11,12]. Nevertheless, the use of CAD-CAM was first restricted to manufacturing inlays and onlays due to the limitations of CAD software. Nowadays, several commercial CAD software systems are available for designing onlay restorations over chair-side procedures [4,7,9,15,16]. The manufacturing process of the onlay is carried out on milling (CAM) a block or disc composed of resin-matrix composite or ceramic [10,15,17,18]. CAD-CAM blocks of resin-matrix composites are industrially polymerized under standard and controlled conditions (temperature and pressure) to producing micro- or nano-scale hybrid resin-matrix composites [7,15,18,19,20]. Thus, the physical and chemical stability of materials manufactured by CAD-CAM are higher than that of traditional manufacturing procedures.

The cementation of onlay restorations over tooth structures is currently performed by using traditional resin-matrix cements [6,21,22,23,24,25]. The polymeric matrix of resin-matrix cements is resultant from a cross-linking of methacrylate-based monomers (at around 22–60 wt%), such as urethane dimethacrylate (UDMA), triethylene glycol dimethacrylate (TEGDMA), bisphenol-A- diglycidylmethacrylate (Bis-GMA), and ethoxylated bisphenol-A dimethacrylate (Bis-EMA) [26,27,28,29]. The inorganic fraction (around 40–80 wt%) of the materials can involve particles of amorphous silica, ytterbium fluoride, zirconium, or barium silicate [5,26]. The combination of monomers and inorganic fillers determines the physical properties of the resin-matrix cements. Thus, resin-matrix cements reveal a chemical composition quite similar to that found in resin-matrix composites for direct and indirect dental restorations [5,26,29,30,31,32,33,34,35,36]. However, significant differences can be noticed on several physical properties, such as strength, elastic modulus, viscosity, and hardness. Recently, flowable resin-matrix composites have been studied as alternative materials for cementation [30,31]. That can bring advantages on the mechanical performance of the interface since resin-matrix composites have higher elastic modulus, fracture toughness, and flexural strength when compared with resin-matrix cements [32,33,36]. The content of inorganic fillers can reach up to around 83 wt% in the chemical composition of the flowable resin-matrix composites [30,31,34]. Inorganic fillers are added at different size and morphological aspects, although currently available resin composites involves a combination of micro- (1–5 µm) and nano-scale (40–60 nm) particles [32,34,35].

The chair-side cementation procedures involve an intrinsic technique sensitivity leading to an irregular layer of the luting material [21,22,23,24,25]. In fact, the viscosity and thickness layer of the interface cement material are the major issues since a minimum resin-matrix cement layer at micro-scale dimension is clinically recommended. Viscosity and thickness of the interface cement material can be influenced by various elements, such as polymeric matrix, inorganic particles, fitting, and degree of conversion of the organic matrix [21,22,23,24,25,37,38,39,40,41]. The layer thickness of the luting material enhances the restoration seating, mechanical performance, and decreases the microleakage and degradation at the restorative margins [21,23,25,27,28]. Several studies recommend a previous conditioning of the onlay inner surfaces with silane and/or methacrylate-based adhesive systems to improve the adhesion to resin-matrix cements and the clinical success of the restoration [21,23,25,27,28]. The low-viscosity of the methacrylate-based adhesive promotes a flowability throughout micro-scale peaks and valleys. The low-viscosity adhesive agent is compressed throughout the micro-scale retentive regions on cementation loading with the resin-matrix cement. Then, a mechanical interlocking occurs between restorative materials, resin-matrix cement, and the substrate after polymerization of the resin-matrix cement. An unproper flowing of the luting material and adhesive system occurs due to the decrease in the cementation loading concerning the chair-side technique sensitivity. That can result in an increased thickness of the cementation layer at the onlay restoration to enamel or dentin interface.

The main aim of the present study was to assess the layer thickness and microstructure of traditional resin-matrix cements and flowable resin-matrix composites at dentin and enamel to composite onlay interfaces after cementation on low loading magnitude. It was hypothesized that the resin-matrix cement or composite layer varies at the onlays to enamel and dentine interfaces depending on the type of material used for cementation as well as on low loading magnitude.

## 2. Materials and Methods

### 2.1. Preparation of Tooth Substrates

Twenty extracted third molars gathered from human participants were first submerged in distilled water for 10 min, then in a solution of 2% sodium hypochlorite (NaOCl) for 10 min. Afterwards, teeth were immersed in 10% formalin solution for 1 week. Finally, teeth were stored in 0.9% NaCl solution for rehydration over a period of 7 days prior to the cementation procedure. The manipulation of extracted third molars was approved by the Human Research Ethics Committee at the University Institute of Health Sciences, cod. 13/CE-IUCS/CESPU/2022, that is in agreement with the Helsinki declaration of 1964. The participants signed the informed consent prior to inclusion in the project since the purpose of the project was described. The participants did not suffer from any systemic diseases and they show worthy oral health, free of antibiotic therapy over the previous 24 weeks.

**Figure 1:**
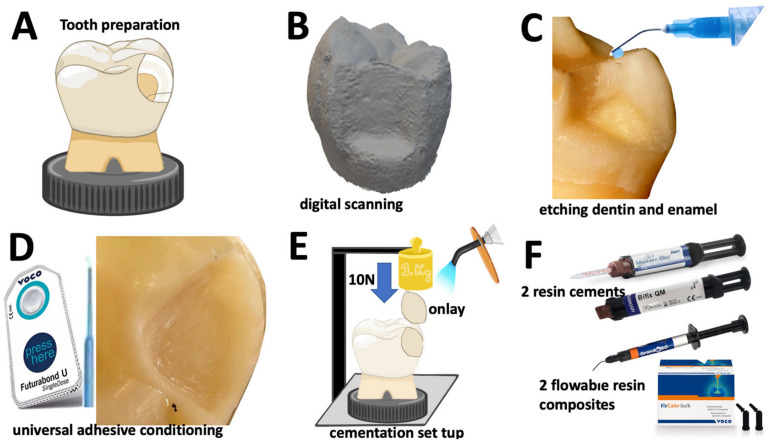
Schematics of the preparation of specimens for optical microscopy. (**A**) Tooth preparation, and (**B**) digital scanning image. (**C**) Total etching procedure, and (**D**) adhesive conditioning on dentin and enamel. (**E**) Cementation on 10 N loading (1 kg weight) through a dental inspector apparatus. (**F**) Resin-matrix cements and flowable resin-matrix composites.

At first, onlay preparation was drawn on each tooth with 4 mm-depth measure from the occlusal cuspid (Figure 1A). Then, a tooth-shaping was performed using taper conical diamond burs following standard guidelines for restorations with onlays. Therefore, onlay shaping involved removal of one occlusal cusp and tooth grounding with 4 mm depth (Figure 1B). Enamel and dentin surfaces presented smooth inner angles and rounded transitional surfaces using spherical diamond burs. All the teeth were sectioned at the root with a taper conical end diamond burs [4,6,37,38,39]. Teeth were mounted in acrylic resin using a dental inspector device (Ney surveyor^TM^, Ney-Tech, Bellevue, WA, USA) to align the pulp floor preparation parallelly to the plan of surface (Figure 1A,E). Tooth surfaces were scanned using a digital scanner (S600 Scanner^TM^, Zirkonzahn GmbH, Gais, Italy) for further milling of the onlays.

On the tooth shaped surfaces, dentin and enamel were etched with orthophosphoric acid (H_3_PO_4_) gel etchant at 37.5% (Optibond gel etchant^TM^, Kerr, Kloten, Switzerland) for 15 s and 30 s, respectively. Then, surfaces were rinsed with air/water jet for 30 s. The excessive amount of water on dentin and enamel was removed using cotton. Lastly, dentin and enamel were conditioned using an universal adhesive (Futurabond M+^TM^, VOCO GmbH, Cluxhaven, Germany) by rubbing with a microbrush for 20 s. An oil-free air was applied onto the adhesive layer for 5 s.

### 2.2. Preparation of Onlay Restorations

Digital scanning was performed for each specimen using the Archiver software^TM^ (Zirkonzahn GmbH, Gais, Italy) to providing CAD files (Figure 1B). The modelling resolution for onlay restoration and the tooth shaped area were carefully customized regarding the morphological aspects for cementation. CAD files were exported as STL files using the Modellier software^TM^ (Zirkonzahn GmbH, Gais, Italy). The STL files and specimens were correlated for further cementation of the onlay restorations, as seen in Figure 1.

Twenty customized onlays were manufactured from resin-matrix composite (GrandioSO^TM^ disc, VOCO GmbH, Cluxhaven, Germany) by CAD-CAM to guarantee precise fitting over each tooth shaped substrate. Onlay lingual cusp tip with dimensions of 2 × 2 mm were designed to contact the stainless-steel counterbody of the dental inspector apparatus and provide an axial loading over the cementation. Onlay materials were milled using a CAM (Imes-icore^TM^, Coritec 250i, Imes-icore GmbH, Eiterfeld, Germany) operated by a software (Hyperdent^TM^, LaserMaq, Aveleda, Portugal). The positioning of the onlays was confirmed using a predictive animation of the milling process prior to the manufacturing. The axial alignment of the onlay specimens over the tooth cavity was achieved using a dental inspector apparatus (Ney surveyor^TM^, Ney-Tech, Bellevue, WA, USA), as seen in Figure 1E. The onlay inner surfaces were grit-blasted with 50 μm alumina (Al_2_O_3_) at 2 bar and 10 mm away from the surface for 20 s. Surfaces were ultrasonically rinsed in isopropyl alcohol for 10 min, then in distilled water for 10 min. The inner surfaces of the onlays were conditioned with a silane compound (Ceramic bond^TM^, VOCO GmbH, Cluxhaven, Germany) over a period of 60 s, then gently oil-free air dried for 5 s following the manufacturer’s instructions [2,6]. Lastly, the inner surfaces were conditioned by a universal adhesive (Futurabond M+^TM^, VOCO GmbH, Cluxhaven, Germany) by rubbing with a microbrush for 20 s. An oil-free air was applied onto the adhesive layer for 5 s to remove any solvents [2,6].

### 2.3. Cementation Procedure and Specimens

On the cementation, two traditional self-adhesive resin-matrix cements were assessed: group M (Max Cem Elite^TM^, KERR, Kloten, Switzerland), and group B (Bifix QM ^TM^, VOCO GmbH, Cluxhaven, Germany). Additionally, a traditional flowable resin-matrix composite, named group G (GrandioSO Heavy Flow^TM^, VOCO GmbH, Cluxhaven, Germany), and a thermally induced flowable resin-matrix composite, named group V (VisCalor bulk- fill^TM^, VOCO GmbH, Cluxhaven, Germany), were assessed in this study for comparison with the resin-matrix cements (Figure 1F and Table 1). Onlay specimens were then assembled to the dental inspector apparatus using an autopolymerizing acrylic resin (Ortho resin^TM^ Dentsply, Chalotte, NC, USA) within a polyvinyl chloride mold to provide a mechanical stability over the cementation procedure. Thus, an optimum restorative fitting was established over the tooth surfaces since the onlay structures were manufactured by CAD-CAM following standard guidelines used in dentistry (Figure 1D,E).

The universal adhesive was not light-cured onto the surfaces before the cementation with the resin-matrix cements and the flowable resin-matrix. Then, the resin-matrix composites were placed onto the inner surfaces of the onlays and fitted onto the tooth cavity. The resin-matrix cements and composites were applied onto each onlay inner surfaces, then placed over the corresponding tooth on 10 N axial loading using a 1 kg weight through the dental inspector apparatus for 60 s, as seen in Figure 1E. The thermally induced flowable resin-matrix composite (VisCalor bulk- fill^TM^, VOCO GmbH, Cluxhaven, Germany) was heated up to 61 °C for 2.5 min using a hand-held dispenser (VOCO GmbH, Cluxhaven, Germany) following the manufacturer’s recommendations to achieve a flowable consistency for cementation.

A silicone key and the dental inspector apparatus were used to ensure a positioning stability avoiding horizontal dislocation of the restoration and tooth on axial loading [24,25]. The excessive cement layer was avoided using a microbrush [2,6], then the luting materials were light-cured under visible light (400–500 nm) using a LED unit (SmartLite Focus^TM^, Dentsply Sirona, Chalotte, NC, USA) at 1200 mW/cm^2^ for 40 s per segment [2,6,40]. The clinical cementation loading was mimicked following previous studies [6,24,41].

**Figure 2:**
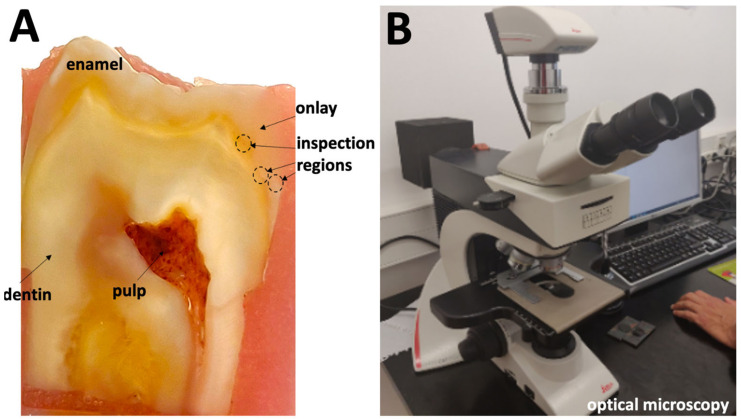
(**A**) Optical micrography at ×10 of the cross-sectioned onlay to dentin and enamel interface. (**B**) Optical microscopy.

After cementation, onlay-to-tooth assemblies were inserted in autopolymerizing polyether-modified resin (Technovit 400; Kulzer GmbH, Wherheim, Germany) in polyvinyl chloride mold [2]. Then, assemblies were cross sectioned at 90 degrees relative to the plane of the restoration pulp floor at the center of the onlay. Specimens were cross-sectioned by wet-griding at low speed using a standard laboratory metallographic machine (Struers, Cleveland, OH, USA) and SiC papers ranging from 120 down to 400 mesh [2]. Surfaces were ultrasonically rinsed in isopropyl alcohol for 5 min, then dried at room temperature. A photomicrography of a cross-sectioned specimen at ×10 is shown in Figure 2A.

### 2.4. Microscopic Analyses

Cross-sectioned specimens were examined by optical microscopy at magnification ranging from ×50 up to ×1000. Microstructural analyses were carried out at different regions along the cement layer interface using a microscope (Leica DM 2500 M^TM^; Leica Microsystems, Wetzlar, Germany) coupled to a computer for image acquisition using a Leica Application Suite^TM^ software (Leica Microsystems, Wetzlar, Germany), as seen in Figure 2B. On each specimen, a total of six micrographs were acquired at ×500 magnification (*n* = 30).

Black and white images were analyzed by using Adobe Photoshop^TM^ (Adobe Systems Software, San Jose, CA, USA). Black regions represented the organic matrix, and the white regions represented the inorganic fillers. The measurement of thickness dimensions of the resin-matrix and adhesive layers was performed from the coronal margins towards the cervical margins along the cement layer interface using Image J^TM^ software (National Institutes of Health, Bethesda, MA, USA) (Figure 2A). The measurement of cement layer thickness was performed perpendicularly to the interface plane, although the interface follows the contours of the tooth and onlay cementation surfaces. Additionally, the mean values of horizontal and vertical discrepancies at the onlay margins were recorded and analyzed among groups. The dimensions of the inorganic fillers were measured parallelly and perpendicularly to the long axis of the particles.

The data was statistically analyzed by normality test Shapiro–Wilk and two-way ANOVA to define statistical differences in the resin-matrix cement thickness values between groups. The cementation thickness layer between groups was compared using the t student test. Additionally, power analysis was performed by t student test to determine the number of specimens for group (n), and to disclose a test power of 100% in this study. Statistical analyses were carried out using Origin Lab statistical software (Origin Lab, Northampton, MA, USA). A probability value <0.05 was considered significant.

## 3. Results

**Figure 3:**
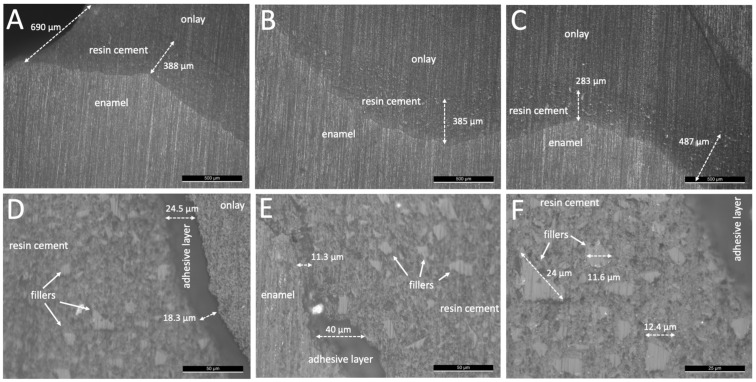
Optical microscopy images of onlay restorations cemented with MaxCem^TM^ resin cement (group M) at magnification of (**A**–**C**) ×50, (**D**,**E**), ×500, and (**F**) ×1000.

**Figure 4:**
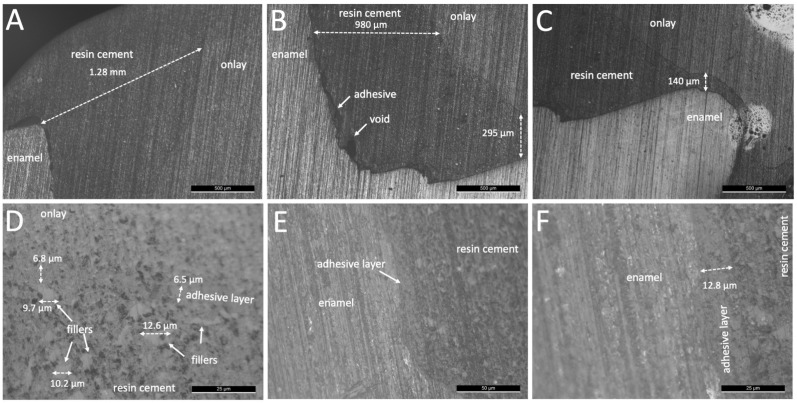
Optical microscopy images of onlay restorations cemented with Bifix^TM^ resin cement (group B) at magnification of (**A**–**C**) ×50, (**D**) ×1000, (**E**) ×500, and (**F**) ×1000.

Optical microscopy images of the onlay interfaces using a resin-matrix cements are shown in Figure 3 and Figure 4. The resin-matrix cement specimens from group M showed a thick layer at the coronal and cervical margin regions, as seen in Figure 3. On the coronal region, the layer thickness values of the resin-matrix cement achieved up to 690 µm, while the mean thickness at the cervical margin was around 480 µm. The layer thickness of the resin-matrix cement ranged from 280 µm up to 690 µm. Additionally, the adhesive layer did not flow on cementation, resulting in an adhesive cement layer thickness ranging from 18 µm up to 40 µm, as seen in Figure 3D–F. On higher magnification images, inorganic fillers were noticed with a mean size ranging from approximately 11 µm up to 24 µm (Figure 3D). As seen in Figure 4, the resin-matrix cement specimens from group B also showed a thick layer at the coronal and cervical margin regions. On the coronal region, the layer thickness values of the resin-matrix cement achieved up to 1.28 mm, while the thickness at the cervical margin showed the lowest mean values of around 140 µm.

**Figure 5:**
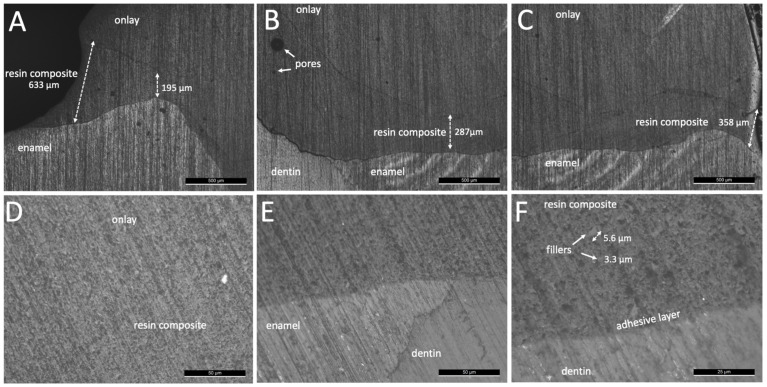
Optical microscopy images of onlay restorations cemented with GrandioSO^TM^ heavy flow resin composite (group G) at magnification of (**A**–**C**) ×50, (**D**,**E**), ×500, and (**F**) ×1000.

**Figure 6:**
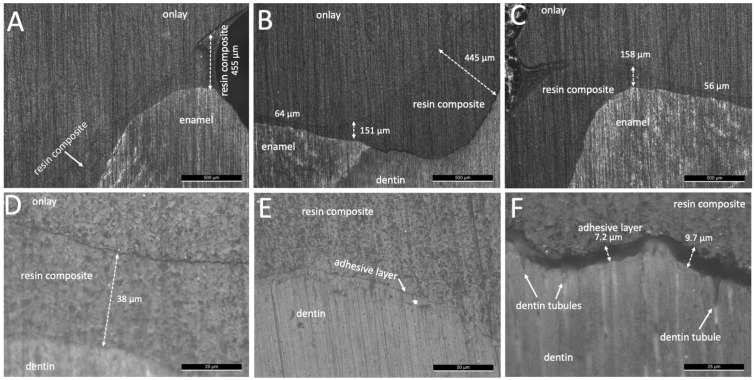
Optical microscopy images of onlay restorations cemented with Viscalor^TM^ flowable resin composite (group V) at magnification of (**A**–**C**) ×50, (**D**,**E**) ×500, and (**F**) ×1000.

Thus, the low loading at 10 N did not provide the fitting of the onlays resulting in a thick resin-matrix cement layer from both groups M and B (Figure 3 and Figure 4). In the same way, the adhesive layer did not flow on cementation resulting in a mean layer thickness value at 12 µm, as seen in Figure 4E,F. On higher magnification images, inorganic fillers were noticed with a size ranging around 9 and 12 µm (Figure 4D). Optical microscopy images of the interfaces of the onlays cemented using flowable resin-matrix composites are shown in Figure 5 and Figure 6. The flowable resin-matrix composite specimens from group G showed a thick layer at the coronal and cervical margin regions, as seen in Figure 5.

On the coronal region, the layer thickness values of the resin-matrix composite achieved up to 633 µm, while the thickness at the cervical margin was around 358 µm (Figure 5). The thickness of the resin-matrix composite layer ranged from 195 µm up to 287 µm. The adhesive layer was also noticed, as seen in Figure 5F. On higher magnification images, most of inorganic fillers were smaller than 6 µm (Figure 5F). In Figure 6, the thickness of the thermally induced flowable resin-matrix composite specimens from group V at the coronal region was recorded at 455 µm, and the cervicall margin interface revealed a thickness of around 158 µm. Low thickness values of the thermally induced flowable resin-matrix composite layer was recorded at 38 µm and 56 µm (Figure 6C,D). The thickness of the adhesive layer was measured at around 12.8 µm, as seen in Figure 6E,F.

**Figure 7:**
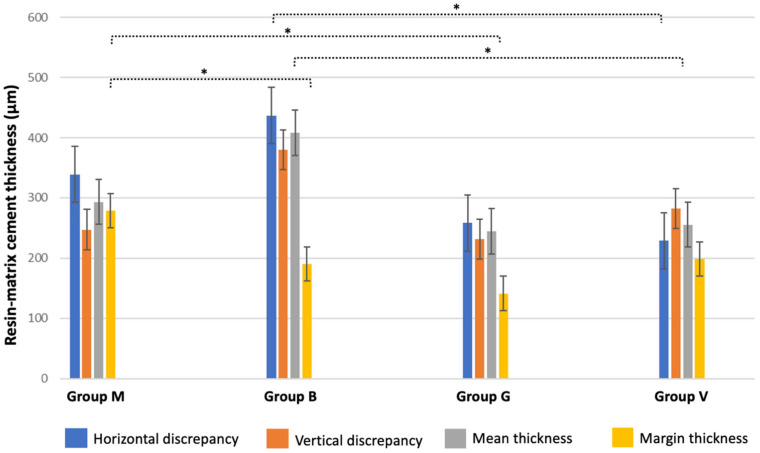
Mean values and standard deviation of the layer thickness and (horizontal and vertical) onlay margins’ discrepancies recorded for resin-matrix cements (M and B) and composites (G and V) at different parameters. Statistical differences were identified as * (*p* < 0.05).

Layer thickness values recorded for resin-matrix cements and flowable composites are shown in Figure 7.

The highest mean layer thickness values of resin-matrix cement were recorded at 405 µm for group B. Statistical differences were detected between group B and the other tested groups (*p* < 0.05). Considering the margins of the onlay restorations, the highest mean layer thickness values of resin-matrix cement were recorded at 285 µm for group M. Statistical differences were noticed between group M and B or G (*p* < 0.05). The highest vertical and horizontal values of resin-matrix layer thickness were recorded at 385 µm for group M and at 425 µm for group B, respectively. Statistical differences in vertical or horizontal thickness were detected between group B and the other groups (*p* < 0.05). There were no statistical differences between the resin-matrix composites.

## 4. Discussion

This study reported a detailed microscopic inspection of the microstructure of resin-matrix cements and flowable resin-matrix composites after cementation of resin-matrix composite onlays to tooth surfaces. Additionally, the thickness measurement of the resin-matrix cement layers and flowable resin-matrix composites was carried out at different regions after cementation on loading. The resin-matrix cement and flowable composites revealed an irregular layer thickness and defects, such as macro-scale spaces and pores. The adhesive showed also a variable layer due to the low flowability of the luting materials. In fact, the results validate the hypothesis of this study. A comprehensive discussion on the main aspects affecting the cementation of resin-matrix cements and flowable composites is fundamental to guide professionals in choosing the type of materials and mode of cementation.

After mechanical preparation and cleaning of surfaces, a conditioning of the surfaces using silane and methacrylate-based adhesive provide a high integrity interface with the resin-matrix cements and flowable composites [2,42,43,44,45,46]. At first, a coating with silane increased the surface wettability of onlay inner surfaces by condensing hydroxyl and SiO_2_ groups. Then, a chemical bonding is established through SiO_2_ and hydroxyl groups on the onlay inner surface. On the cementation, a chemical reaction occurs between free radicals in the monomers’ matrix and the hydroxyl and SiO_2_ groups to establish a chemical bonding to the resin-matrix luting materials [36]. Micro-scale irregularities on the tooth and onlay inner surfaces can be filled by conditioning with low-viscosity methacrylate-based adhesives, including the universal adhesive used in the present study. Then, rough surfaces are coated with the adhesive layer and resin-matrix cement establishing a mechanical interlocking after polymerization [47,48]. A relatively viscous resin-matrix cement or flowable composite could not reach the deepest micro-scale valleys on the surface without the adhesive layer. A low flowing of a resin-matrix cement and flowable composite promotes the formation of pores or voids, and the lack of mechanical retention. Then, an absence of adhesive between indirect restorations and resin-matrix cement or composites could decrease the bond strength of the interface [6,21,24,49,50]. Another issue is related to the application of low-viscosity adhesive by using a hand-held micro-brush under reciprocating sliding (rubbing movement) onto the surfaces for 20 s. The adhesive conditioning has an intrinsic sensitivity concerning the operator-induced factors, such as movement, load, time, air drying, and amount. Consequently, the layer thickness of adhesive also varies depending on the application mode, surface conditions, and type of adhesive. In this study, the layer thickness of the universal adhesive system ranged from 12 µm up to 40 µm for the resin-matrix cements while a layer thickness of around 7–12 µm was detected for the flowable resin-matrix composites. As seen in Figure 4 and Figure 6, a considerable layer of low-viscosity methacrylate-based adhesive was accumulated at certain regions of the surfaces, probably due to the adhesive amount and low loading cementation. On the physical properties, the adhesive layer is the most mechanically susceptible material at the interface, and therefore, mechanical failures can take place by stresses under polymerization shrinkage, mastication loading (1–500 N), or thermal oscillations (i.e., 5–50 °C). The mean values of elastic modulus and strength of the adhesive system are lower than those recorded for the resin-matrix cement and flowable resin composites (Table 1).

As seen in optical microscopy images, the highest layer thickness values of resin-matrix were measured for the resin-matrix cements. Indeed, the low loading cementation negatively disturbed the flowing of the resin-matrix cements and fitting of the onlay restoration. On the contrary, the thermally induced and the traditional flowable resin-matrix composites revealed a proper flowing considering the lower values of layer thickness after low loading cementation. In clinical situations, cementation procedures at low loading can occur since it depends on the professional expertise and chair-side sensitivity. A previous study measured the cementation pressure applied from different dentists and the loading values ranged from 12 N up 67 N [51]. In previous studies, the fitting and strength of the interface are enhanced when the cementation loading increased [49,52]. In the present study, the authors assessed the restorative interface when the cementation loading occurred at low magnitude. The loading magnitude assessed in this study corroborates with the values reported in the literature. However, an increased layer thickness of resin-matrix cement or flowable composites also increases the formation of defects, such as micro- and macro-scale pores and spaces, as seen in Figure 4 and Figure 5. Thus, marginal discrepancy dimensions at indirect restorations to tooth surfaces should be less than 100 μm [52].

The thickness of the cementation layer can be affected by the inorganic filler content, the filler size, organic matrix components, materials’ viscosity, and the polymerization reaction [5,21,26]. The traditional flowable resin-matrix composites revealed an adequate viscosity, and therefore they can flow on low loading cementation. A pre-heating of thermally induced flowable resin-matrix composites enhanced the monomer mobility leading to a higher overall flowing and degree of conversion of monomers. The size of inorganic fillers of a resin-matrix cement was detected at approximately 35 μm that determined its minimum layer thickness. Additionally, the size of the micro-scale inorganic fillers at high content (67 wt%) decreases the viscosity of the resin-matrix cement. The flowable resin-matrix composites possess micro- (1–3 μm) and nanoscale (40–60 nm) dimensions at high content (83 wt%) that avoided a large cementation layer, as seen in Table 1.

The present in vitro study revealed a detailed microscopic analysis of onlay restorations cemented to dentin and enamel surfaces using resin-matrix cements and flowable composites. However, limitations are related to the technical sensitivity of the adhesive conditioning and cementation procedures, even though a single operator performed the preparation of specimens. Indeed, the loading and mode of cementation are dependent on the operator. The onlay fitting to the tooth shaped substrate depends on the digital scanning resolution and processing of onlays. This study focused on the use of resin-matrix composite onlay prepared by using a standard CAD-CAM protocol although the assessment of lithium disilicate or zirconia could be interesting for comparison with the composite blocks regarding the prosthetic fitting, surface conditions, and the layer thickness of the resin-matrix cement and flowable composites. The treatment of the onlay inner surfaces was performed only by grit-blasting using alumina particles, then conditioning with silane compounds and methacrylate-based adhesives. The increase in roughness of the onlay inner surface also increases the mechanical interlocking of the adhesive and resin-matrix cement or flowable composite. Several types of adhesive systems and resin-matrix cements should be assessed since their chemical composition and physical properties determine the mechanical interlocking, the cementation layer thickness, and the mechanical integrity of the interfaces. Considering the cementation, the loading can vary and therefore it should be correlated with different resin-matrix cement and flowable composite. The polymerization procedures should be controlled regarding the equipment conditions, mode, and exposure time. In fact, the cementation procedures of prosthetic structures are not well-controlled due to the clinical sensitivity, and therefore, all the above-mentioned variables can influence the long-term success of onlay restorations.

## 5. Conclusions

Within the limitations of this study, the main concluding remarks can be drawn as follows:

- A higher resin-matrix layer thickness was found for resin-matrix cements than that recorded for flowable resin-matrix composites after cementation at a low loading magnitude. The layer thickness of the resin-matrix cements and flowable composites varied along the onlay to dentin and enamel. An increased cementation layer thickness is more vulnerable to the formation of defects, such as macro- and micro-scale voids and pores.
- The adhesive layer also varied at the interfaces due to the lack of flowing of the resin-matrix cement and flowable composites on cementation at low loading magnitude. Additionally, an increased layer thickness of adhesive and resin-matrix cement or flowable composite can negatively affect the mechanical integrity of the interface since those materials reveal the lowest records of mechanical properties, such as strength, elastic modulus, and fracture toughness.
- Cementation procedures on low loading can occur in several clinical situations due to the operator technical sensitivity. An increase in the cementation loading magnitude by high handling pressure could promote a proper flowing of the adhesive system and the resin-matrix cements resulting in an adequate layer thickness at the onlay interface.

## Figures and Tables

**Table 1:** Details of the materials used in this study.

Material Type (Brand, Manufacturer)	Organic Matrix	Fillers %(*w*/*w*)	Fillers % (*v*/*v*)	Filler Type	Elastic Modulus (GPa)
Dual-curing resin cement (Max Cem Elite^TM^, KERR Kloten, Switzerland)	Bis-GMA, HEMA, GPDM; UDMA; 1,1,3,3-tetramethylbutyl hydroperoxide TEGDMA, CHPO, MEHQ Bis-GMA, GPDM, co-monomers (33 wt%)	67	46	Barium alumina silica glass, fluoroalumina silicate glass borosilicate (30–60%) glass, Ytterbium fluoride (10–30%), amorphous silica (1–5%). (size~3.6 µm).	4.5
Dual-curing, resin cement (Bifix QM^TM^, VOCO GmbH, Cluxhaven, Germany)	Bi-functional methacrylate, acid methacrylate, Bis-GMA, benzoyl peroxide, amines and BHT, Gly-DMA, UDMA, phosphate monomers (30 wt%)	70	61	Glass fillers, amorphous silica; (size~2.9 µm)	6–7.5
Flowable resin composite (GrandioSO Heavy Flow^TM^, VOCO GmbH, Cluxhaven, Germany)	BisGMA, BisEMA, TEGDMA, HDDMA, CQ, amine and BHT (17 wt%)	83	68	nanoparticles of SiO_2_ (size~20–40 nm); glass-ceramic; (size~1 µm)	11.5
Thermally induced flowable resin composite (VisCalor bulk- fill ^TM^, VOCO GmbH, Cluxhaven, Germany)	Bis-GMA, aliphatic dimethacrylate (17 wt%)	83	68	nanoparticles of SiO_2_ (size~20–40 nm); glass-ceramic (size~1 µm)	12.3–17.5

## Data Availability

The datasets used and/or analysed during the current study available from the corresponding author on reasonable request.
